# Designs of two randomized, community-based trials to assess the impact of alternative cookstove installation on respiratory illness among young children and reproductive outcomes in rural Nepal

**DOI:** 10.1186/1471-2458-14-1271

**Published:** 2014-12-15

**Authors:** James M Tielsch, Joanne Katz, Scott L Zeger, Subarna K Khatry, Laxman Shrestha, Patrick Breysse, William Checkley, Luke C Mullany, Steven C LeClerq

**Affiliations:** Department of Global Health, Milken Institute School of Public Health, George Washington University, Washington, DC, USA; Department of International Health, Johns Hopkins Bloomberg School of Public Health, Baltimore, MD USA; Department of Biostatistics, Johns Hopkins Bloomberg School of Public Health, Baltimore, MD USA; Nepal Nutrition Intervention Project – Sarlahi, Kathmandu, Nepal; Department of Pediatrics and Child Health, Institute of Medicine, Tribhuvan University, Kathmandu, Nepal; Department of Environmental Health Sciences, Johns Hopkins Bloomberg School of Public Health, Baltimore, MD USA; Department of Medicine, Johns Hopkins School of Medicine, Baltimore, MD USA

**Keywords:** Pneumonia, ALRI, Birthweight, Biomass fuel, Household air pollution, Improved cookstoves, Randomized trials

## Abstract

**Background:**

Acute lower respiratory infections (ALRI) are a leading cause of death among children. Low birthweight is prevalent in South Asia and associated with increased risks of mortality, and morbidity, high levels of indoor household air pollution caused by open burning of biomass fuels are common and associated with high rates of ALRI and low birthweight. Alternative stove designs that burn biomass fuel more efficiently have been proposed as one method for reducing these high exposures and lowering rates of these disorders. We designed two randomized trials to test this hypothesis.

**Methods/design:**

We conducted a pair of community-based, randomized trials of alternative cookstove installation in a rural district in southern Nepal. Phase one was a cluster randomized, modified step-wedge design using an alternative biomass stove with a chimney. A pre-installation period of morbidity assessment and household environmental assessment was conducted for six months in all households. This was followed by a one year step-wedge phase with 12 monthly steps for clusters of households to receive the alternative stove. The timing of alternative stove introduction was randomized. This step-wedge phase was followed in all households by another six month follow-up phase. Eligibility criteria for phase one included household informed consent, the presence of a married woman of reproductive age (15–30 yrs) or a child < 36 months. Children were followed until 36 months of age or the end of the trial. Pregnancies were identified and followed until completion or end of the trial. Phase two was an individually randomized trial of the same alternative biomass stove versus liquid propane gas stove in a subset of households that participated in phase one. Follow-up for phase two was 12 months following stove installation. Eligibility criteria included the same components as phase one except children were only enrolled for morbidity follow-up if they were less than 24 months.

The primary outcomes included: incidence of ALRI in children and birthweight.

**Discussion:**

We presented the design and methods of two randomized trials of alternative cookstoves on rates of ALRI and birthweight.

**Trial registration:**

Clinicaltrials.gov (NCT00786877, Nov. 5, 2008).

## Background

Acute lower respiratory infections (ALRI) remain a leading cause of death among children in the developing world with the highest risk among the youngest children [1]. Small size at birth, including low birthweight (LBW) is also common and an important risk factor for early infant death with 28% of all neonatal deaths attributable directly to this condition [2]. Over the past few decades, there has been an increasing focus on the role of environmental exposures in the etiology or exacerbation of ALRI. In the mid-1950s in London, thousands of excess deaths, especially among young children and the elderly, were associated with extensive outdoor particulate air pollution due to smoke from coal burning household stoves [3]. More recent studies of outdoor air pollution have confirmed that respirable particulate exposure, even at the relatively low levels now seen in high-income settings, can increase respiratory mortality and morbidity [4–6]. While outdoor exposures in low- and middle-income countries play a role in the incidence and severity of respiratory disease [7], outdoor exposures are most extreme in urban environments and the vast majority of the studies on outdoor air pollution from developing countries have been done in these settings. Of more concern in rural areas is indoor air pollution where particulate matter concentrations can be many times those found outdoors [8]. Such high levels are particularly frequent in south Asia where biomass materials (dung, wood, and crop residue) are the predominant sources of fuel used for cooking and heating and where typical houses have limited ventilation and use open stoves without flues. It is not uncommon to observe concentrations between 2,000 and 15,000 μg/m^3^ during cooking [8–10], but very high exposures can continue throughout the day if the stove is also used for heat or the family prepares food items for sale. The observational epidemiological literature on the association of indoor smoke exposure and ALRI in young children demonstrates that exposure to incomplete combustion of biomass fuels increases by 2–12 fold the risk of respiratory symptoms or disease in preschool age children [11].

It is well known that certain particulate matter and other chemical respiratory exposures can cause reduced birthweight, intra-uterine growth restriction (IUGR), and preterm birth; the best examples being active and passive tobacco smoke exposure during pregnancy [12]. The observational evidence on the association between indoor air pollution and infant birth outcomes suggests that exposure to biomass fuel smoke is associated with a 97 g reduction in birthweight but the depth and quality of this literature varies substantially [13].

Other chronic disease outcomes are also associated with poor indoor air quality and the global burden of illness due to indoor burning of biomass fuels in 2010 has been recently estimated at 4.3% of all disability adjusted life years (DALYs) [14]. Use of cookstoves that utilize alternate fuels or incorporate a chimney for ventilation may reduce exposure and thus improve outcomes, but neither the exposure reduction nor the concomitant health gains have been adequately studied at the population level. We have implemented trials in rural Nepal to examine these questions, and present here the design of those trials.

## Methods

### Design

The Nepal Cookstove Intervention Project is a pair of randomized trials to evaluate the impact of using alternative cookstoves to improve indoor air quality, respiratory morbidity in young children, and reproductive outcomes in a population where indoor open burning of biomass fuels sources are the norm. The study was initially designed as a single trial with two identical cohorts, but after examining the exposure reduction performance of the alternative stove used in the first trial, it was decided to alter the second phase to a separate, but complementary randomized trial.

**Figure 1:**
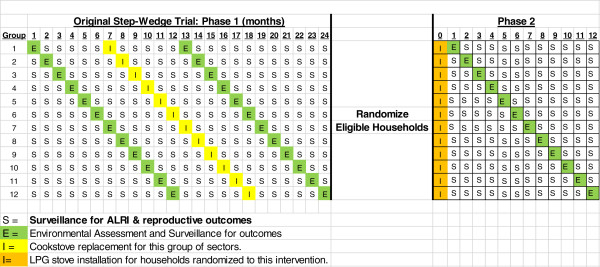
**Trials design scheme.**

Phase One: This was a cluster-randomized, modified step-wedge trial (Figure 1). The study area was initially divided into 51 sectors of 20–30 households that were each estimated to provide between 25 and 40 children < 36 months of age. These sectors served as units for concurrent installation by a single stove installation team of the alternate biomass stove over a one month period. Sectors were then combined into 12 groups that served as the unit of randomization and corresponded to the 12 months of the step-wedge portion of the design. Within a group, sectors were geographically spread across the study area using a stratified systematic sampling procedure in order to prevent an additional level of clustering and within group correlation of the primary outcomes.

This standard step-wedge design was modified by adding a six month period of outcome assessment in all groups prior to the start of the step-wedge and a similar six month period of outcome assessment following completion of the 12 month step wedge. This provided additional person-time and balance on season both before and after new stove installation. All three primary outcomes show marked seasonality in this population [15, 16].

Phase Two: The design of phase two was an individually randomized trial at the household level (Figure 1). Eligible households that participated in phase one and newly eligible households that did not participate in phase one were randomized to either an alternate biomass stove with chimney or a LPG stove with gas tank and 12 month supply. Follow-up of phase two was for 12 months following stove installation.

### Study population

The study population for Phase 1 consists of residents of all households in four Village Development Committees (VDCs) in Sarlahi District in the terai region of southern Nepal. The study population for Phase 2 included only households in 2 of these VDCs. The terai lies along the border with north India and is part of the flood plain of the Ganges River and its tributaries that drain from the Himalayas to the Bay of Bengal. This is not a high mountain area, but one characterized as low and flat (approximately 600 ft. elevation) with high population density. It is typical of most of northern India and large parts of western Bangladesh and northern and central Pakistan with a population of over one billion. It is predominately an area of traditional, rural, Hindu culture. The population is mostly peasant farmers (58%) or laborers (26%) and their families, and is considered a poor area, even in Nepal. It is an extremely poor environment with a per capita income of $146 [17].

### Household eligibility phase 1

Households were eligible for participation in the first trial if they:

- ● Did not currently have an improved, ventilated biomass cookstove, LPG cookstove or electric cookstove in the household.
- ● Used their traditional open burning biomass cookstove indoors or in an area contained by at least 3 walls.
- ● Had household walls of mud, brick, cement, or wood. Thatch or bamboo walled houses that were not plastered with mud were not eligible for fire safety reasons.
- ● Were willing to have us install an alternative, ventilated biomass cookstove and be willing to use it for at least 12 months.
- ● Have a married woman 15–30 years of age or at least one child <36 months of age residing in the household at the time of trial initiation.

### Household eligibility phase 2

Households were eligible for participation in the second trial if they:

- ● Resided in two of the original four VDCs from the Phase 1 trial.
- ● Participated in the first trial and continued to have a married woman age 15–30 years of age or at least one child <24 months of age residing in the household at the initiation of Phase 2.
- ● Were a new household in the study VDCs that had at least one married woman age 15–30 years of age or at least one child <24 months of age residing in the household at the initiation of Phase 2.
- ● Were willing to be randomized to either continuing with their alternative, ventilated biomass stove from Phase 1 or to an LPG stove and to use it for 12 months.

### Intervention

**Figure 2:**
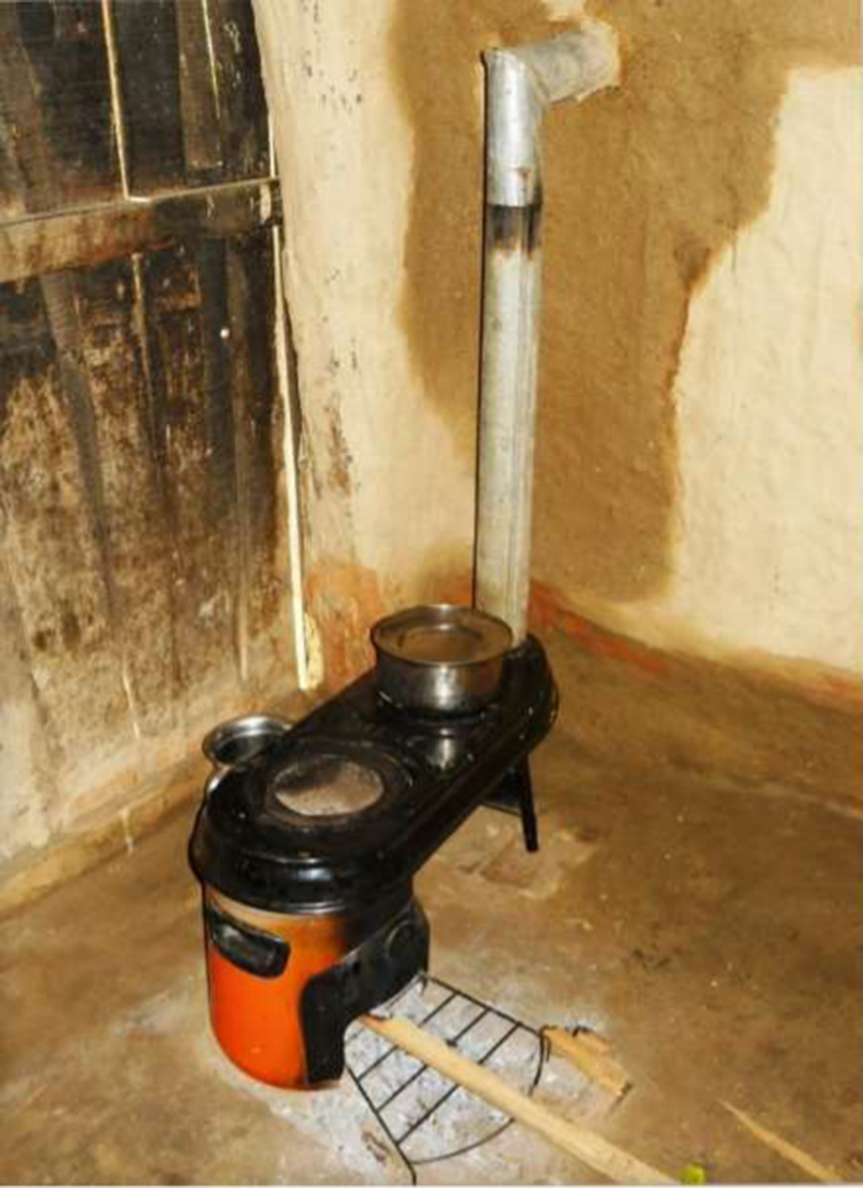
**Alternative biomass cookstove used in both trials.**

Phase 1: The cookstove intervention in the first phase consisted of replacing the household’s traditional open burning stove with a commercially available two burner biomass stove with a chimney outlet and a chimney constructed from sheet metal. The stove used was model G-3300 with the G-3355 two pot attachment manufactured by Envirofit International (Colorado Springs, CO, USA) (Figure 2). Chimney pieces were manufactured by a local sheet metal firm and installed on-site by project stove installation teams. In addition to the provision of a new stove, extensive training and demonstration in the use and maintenance of the stoves was conducted and reinforced regularly throughout the follow-up period by a special team of stove monitors***.***

Phase Two: The cookstove intervention in phase two consisted of either, the continued placement and support for use of the alternate biomass stove (Envirofit model G-3300 + G-3355 with chimney), or the installation of an LPG two-burner stove with gas tank and adequate LPG supply for 12 months. The LPG stoves were manufactured in India and the LPG was sourced from one of three suppliers in Nepal and distributed monthly. All households who were randomized to the improved biomass stove arm were provided with a LPG stove, gas tank, and one month of LPG fuel at the end of the study.

### Primary and secondary outcomes

Primary outcomes for both trials included:

1. Incidence of ALRI among children < 36 months of age. An episode of ALRI was defined as two or more consecutive days with maternal report of fever and fast/difficult breathing with both symptoms reported on at least one day during the episode and during which at least one symptom occurred each day. Consecutive episodes had to be separated by a minimum of 7 symptom free days. An episode of severe ALRI was defined as an episode of ALRI plus one or more of the following:
  ● •respiratory rate >50 if the child was 12 months or older; >60 if the child was younger than 12 months;
  ● •chest retractions;
  ● •unconsciousness;
  ● •chest auscultation finding of crackles in at least one quadrant.

Respiratory morbidity was assessed during weekly household visits where mothers or other caregivers were asked about signs and symptoms on each day in the preceding week. Maternal report of signs and symptoms included high fever, persistent cough, fast or difficult breathing, wheezing, watery stool, ear discharge, and burn injury. We also asked whether the child had been taken for care and where the child was taken. If a child had an episode of fast/difficult breathing in the past week, a separate visit was scheduled for examination as soon as possible by a more senior staff member to assess the child for danger signs, measure respiratory rate, oxygen saturation and conduct digital chest auscultation.

2. Birthweight as measured by study staff using digital infant scales (Tanita Corp., Tokyo, Japan).
3. Gestational age as measured by the start of last menstrual period.

Secondary outcomes for both trials included:

1. Forced expiratory volumes among married women 15–30 years of age and one other randomly selected adult (15 yrs or older) member of the household. Forced expiratory volume in one second (FEV_1_) and forced expiratory volume in six seconds (FEV_6_) were measured using the Piko-6 pocket spirometer (nSpire Health, Longmont, CO, USA) and a disposable mouthpiece under a standardized protocol.
2. Respiratory symptoms as assessed using a modified version of a standardized questionnaire [18] among married women 15–30 years of age and one other randomly selected adult (15 yrs or older) member of the household.
3. Blood pressure and pulse among married women 15–30 years of age and one other randomly selected adult (15 yrs or older) member of the household. Blood pressure was measured using an automated osscilometric device (BpTrue 300, VSM MedTech Ltd, Coquitlam, Canada).
4. Oxygen saturation, carboxyhemoglobin level, and heart rate during pregnancy using a pulse oximeter (Rad-57. Masimo Corp., Irvine, CA).
5. Indoor air concentrations of respirable particulates (PM_2.5_) and carbon monoxide (CO).

### Randomization

Phase One: Randomization of the 12 groups to the timing of new stove installation was conducted in the project office in Kathmandu and was a transparent process with participation of senior field representatives. The numbers 1 through 12 were written on separate slips of paper and senior Nepal-based project staff drew slips one at a time out of a hat with the first group selected being the group that would have their stoves replaced in the first month, the second group selected having stoves replaced in the second month, etc.

Phase Two: Eligible households in phase two were individually randomized stratified by VDC and Ward using the sample procedure in STATA (StataCorp LP, College Station, TX). Households that had participated in phase one were randomized separately from newly eligible households.

### Data collection

The timing of data collection activities is outlined in Table 1 and described in more detail below.

**Table 1:** **Data collection schedule**

	Household enrollment	Pregnancy enrollment	Monthly during pregnancy	Day of birth	One week post-partum	Weekly	Monthly	6 Monthly	End of follow-up
Household roster	**X**								
Household SES & characteristics	**X**								
Pregnancy surveillance							**X**		
Pregnancy history & maternal anthropometry		**X**							
Maternal morbidity		**X**	**X**						
Maternal Blood Pressure		**X**	**X**	**X**	**X**				
Maternal carboxyHb, O_2_ saturation & pulse		**X**	**X**						
Birthweight				**X**					
Child anthropometry	**X**							**X**	
Child vital status				**X**		**X**			
Morbidity						**X**			
Danger signs						**X** ^*^			
Temperature						**X** ^*^			
Respiratory rate & O_2_ saturation, pulse						**X** ^*^			
Chest Auscultation						**X** ^*^			
Adult Respiratory Symptoms & FEV1 and FEV6	**X**								**X**
Compliance with new stove use						**X**			

*For children receiving ALRI case examination.

### Household census

A census of all households in the study area was conducted as a part of phase 1 in order to identify all eligible households for recruitment, obtain informed consent for participation, address eligible households for ease of identification during the data collection phase, and to collect basic information about residents of eligible households. A full household roster was collected and included identifying information, age, date of birth, sex, marital status, literacy, years of education, occupation, tobacco use, weight, height (length for children < 2 yrs), and individual consent status for all household residents. For married women 15–30 yrs and one randomly selected other adult (≥15 yrs), the following additional measurements were collected: FEV_1_, FEV_6_, blood pressure, and heart rate. These people also completed a respiratory symptoms questionnaire.

Assessment of household characteristics was collected for all eligible consented households. Variables collected included measures of household socioeconomic status using an asset index, ethnicity, number and types of cookstoves, house construction materials and size, source of drinking water, household sanitation practices, and whether there were members of the household working outside the local area or country (as a measure of additional cash income).

### Pregnancy identification and data collection

Initial identification of pregnant women occurred at the baseline enrollment visit. Subsequently, all married women between 15 and 30 years of age were visited every 5 weeks and asked about their menstrual period in the preceding 5 weeks. If the woman had her period, the week of the period was recorded and she was visited again 5 weeks later. If she reported no menstrual period, she was offered a urine-based pregnancy test at her home. If the test was positive, she received a pregnancy enrollment interview that included her reproductive history, morbidity in the previous month, date of her last menstrual period, tobacco and alcohol use, and plans for where and by whom she would deliver her baby. We also measured her weight, height, blood pressure, pulse, oxygen saturation, and carboxyhemoglobin level. Pregnant women were encouraged to attend antenatal care clinics in the public or private sector and to deliver at a certified birthing facility. The study provided education on nutrition, clean delivery, and danger signs at this visit. We also provided pregnant women with 90 days of iron-folic acid supplements, a clean birthing kit (included a clean blade, string, plastic tarp, and small plastic disc on which to cut the cord), chlorhexidine ointment for umbilical cord antisepsis, and a single dose of albendazole.

We visited all women monthly throughout their pregnancy to collect pregnancy-related morbidity in the previous month, to ask about tobacco and alcohol use, and to measure weight, blood pressure, pulse, oxygen saturation, and carboxyhemoglobin levels.

### Pregnancy outcome assessment

When a baby was born, the family contacted a local staff member as soon as possible, often during labor. Project staff then visited the home to collect extensive information on the birth process and the health of the mother and newborn infant(s). Birthweight, length, head circumference, and temperature of the infant(s) were measured and recorded. One week following delivery, a visit was made to record information on infant and maternal post-partum morbidity.

### Respiratory morbidity assessment

All eligible children (0 through 35 months of age) were visited weekly for morbidity assessment as described previously. Once a child reached 36 months of age he/she was discharged from the study.

### Cause of death assessment

All women and children who died were identified and a verbal autopsy was conducted with members of the family after a culturally appropriate mourning period.

### Environmental assessment

Each household received a measurement of indoor air quality once prior to new stove installation and once afterwards. This assessment consisted of measurements of PM_2.5_, CO, and temperature and humidity over an 18 hour period that included all the cooking events during the day. All measurements were taken using logging devices set to record every 10 seconds. The instrument used for airborne particulate concentration was the DataRAM pDR 1000AN (Thermo Fisher Scientific Inc., Waltham, MA). This instrument provides particle mass concentration in units of μg/m^3^ and was used in passive mode as a nephelometer. As nephelometric readings vary according to relative humidity, values were corrected based on calibrations with gravimetric measurements as previously described [19]. The instrument for measuring CO concentration data (in units of ppm) was the EL-USB-CO300 (Lascar Electronics Ltd, Salisbury, UK). Temperature and humidity were measured using a HOBO data logger (Onset Computer Corp., Bourne, MA, USA).

### Data management and statistical analysis plans

Interview-based data were collected on paper forms and checked for errors and missing values before being sent from the field site to Kathmandu for data entry. Data entry programs checked for inconsistencies and out of range values. Errors were corrected by data supervisors based on all relevant forms or sent back to the field for clarification if necessary.

### Statistical analysis

The first step in the statistical analysis is calculation of descriptive statistics for exposure, demographic and health outcome data to elucidate basic features in the data, to identify gross outliers and to examine bivariate relationships between pairs of variables. We then proceeded with exposure modeling and health effects analysis at both the individual and village levels for ALRI and birthweight. Throughout, standard software packages such as SAS and R were used, as appropriate.

### Exposure modeling

The raw exposure PM_2.5_ and CO data were pre-processed as follows. First, 10 minute average values were calculated from the 10 second data using 3% trimmed means to avoid undue influence of outliers. The 10 minute averages were then filtered using running medians of length 5 to eliminate short-term excursions caused by larger particle contamination. A base-line value was estimated using the 10th percentile of all measurements for a day. We then defined Stove Influenced Time (SIT) to be any 10-minute interval for which it’s filtered value exceeded the baseline by a multiple of more than 1.2. We defined the Stove Influenced Particulate (SIP) or CO (SIC) as the total difference between the 10-minute average value and the baseline, over the SIT times for a 24 hour period.

For each exposure measure, we used a linear mixed effects regression model as defined below to estimate the average difference between households with an improved stove as compared to control households without one, while controlling for season and secular trend. Because the effect of the stove on indoor air quality can vary by season, we considered two measures: (1) the average household exposure for the year after stove installation as compared to the annual average before; and (2) the average change in exposure immediately after stove installation was done as compared to immediately before. The former captures the season-specific effects of stoves but can be biased by secular trends. The latter is less biased, but more variable and more dependent on the adjustment for seasonal and secular trends.

### Health effects modeling

To estimate the effect on ALRI of having a new cook stove installed in the home, while fully accounting for the salient features of the stepped wedge trial design [20] and for sources of variation at both the individual and village levels, we used a generalized mixed effects model as summarized in Hussey and Hughes [20]. Let *Y*_*ijt*_ denote a positive ALRI response for individual *j* = 1,…,*N* from village *i* = 1,…,*I* at time point *t* = 1,…,*T*. Let *p*_*ijt*_ denote the risk of ALRI, that is the expected value for *Y*_*ijt*_. We assume *p*_*ijt*_ satisfies the logistic random effects model:
1

where: *α*_0_ is the overall baseline log odds of having ALRI;  is the random effect for cluster *i* to account for correlation among children from the same village*;* is the random effect for child j in cluster *i* to account for possible within-child-level correlation; *s*(*t*, *v*) is a fixed, smooth function of time to control for secular and seasonal trends; and *θ* is the fixed cook stove intervention effect that is of primary scientific interest. In this model, we will represent *s*(*t*, *v*) by natural cubic splines with *v* degrees of freedom. A priori, with two years of data, we chose *v* = 8 (4 degrees of freedom per year) but will conduct sensitivity analyses to determine whether the major findings are sensitive to this choice over a range of reasonable choices *v* = 4 to 12 for this smoothness constraint. The generalized linear mixed model above will be estimated using the method of maximum likelihood as implemented in the R package *glmer (*http://cran.r-project.org/web/packages/lme4/lme4.pdf*)*. Robust variance estimates of the stove effect coefficient will be calculated and compared to the model based values as a sensitivity analysis to check on the assumed correlation model within children and among children within villages. If the robust variances are significantly larger than their model-based counterparts, the former will be used for testing and confidence interval calculations.

A similar approach will be used to estimate the treatment effect on birthweight. While seasonality of birthweight and other reproductive outcomes is present in this population, it is not as dramatic as seen for ALRI, nor is the clustering by sector as high [16]. Birthweight will be treated as both a continuous variable and as dichotomous with low birthweight defined as weights <2500 g.

### Sample size

The power of the study to test the null hypothesis that the stove installment effect is zero (*θ* = 0) for the ALRI outcomes was done as follows. We adopted the design represented in Figure 1 with 12 groups of sectors and explain the sample size calculation using the notation from the statistical plan in the previous section. We set the true value the stove effect to take values between *θ* = −0.1 to −0.3 corresponding to 10 to 30% reductions in the odds of ALRI attributable to installation of the clean cook stove. To estimate the power, we specify the parameters of the design and study population as via the following assumptions:

1. The number of eligible children per sector follows a Poisson distribution with mean 35.
2. The ALRI rate will be approximately 1.0 episodes per child per year, conservatively lower than the value 1.3 observed in previous studies in the same area [15].
3. There will be 4 sectors in each of the 12 groups.
4. Seasonality is represented by a sinusoid with relative amplitude of 1.05 so that the peaks and troughs have ALRI rates that are 1.05 times higher or lower than the long-term average.
5. There are two cases for secular trend: (5a) no secular trend; (5b) a large trend that starts at time 1 with a 50% increase in the odds of ALRI and decreases to 0 over 10 months.
6. The random effects variances for village and child are 0.025 and 0.10 respectively. These values imply that villages can have ALRI rates that vary by ±30% (2*√.025) across the population while individuals within a village have rates that vary by ±60% (2*√.1).

To estimate power, we conducted a simulation study in which we first generated a random data set given the parameters described above, then estimated cook-stove affect parameter using the generalized linear mixed effects model specified in Equation (1) above. The simulation was run 100 times for each situation and the estimated power is reported in the table below with standard error of the power less than 5%.

The degrees of freedom *v* used in specifying the time function *s*(*t*; *v*) was allowed to vary between 0 (no secular trend) to 8 (4 per year). When the data are generated without a secular trend, all three values for *v* produce consistent estimates of the cook stove effect. However, when in the case where there is a sizeable trend, the *v* = 2,8 models give consistent estimates, but the model that ignores the trend produces strongly biases estimates. The simulation study shows that the design has adequate poser to detect a decrease of 20% or greater relative to baseline (Table 2).

**Table 2:** **Power to compare ALRI incidence before and after alternative biomass cookstove installed for varying relative odds and degrees of trend in ALRI rates over time**

	True cook-stove effect (θ)								
	(Relative odds)								
Degree of trend in ALRI incidence	−0.1			−0.2			−0.3		
ν	0	2	8	0	2	8	0	2	8
No trend	88	26	34	100	81	89	100	99	99
Large trend	100	19	37	100	80	87	100	99	99

Based on previous research in this study area, we originally expected approximately 6000 live births in the study area for these trials over 4 years. However fertility declined significantly since that previous research was conducted and a modified expectation was in the range of 3000 live births. Approximately 1% died prior to our being able to weigh them leaving approximately 2350 live births in phase one and 600 in phase two. The mean birthweight in our last trial was 2700 grams with a standard deviation of 437 grams [21]. The intra-sector correlation for birthweight was estimated to be 0.03357. Assuming 80% power and a Type I error of 5% (2- sided), the minimum detectable mean difference in birthweight was approximately 57 g in phase 1 and 83 g in phase two. These detectable differences in mean birthweight are in the range of those reported for infants whose non-smoking mothers are exposed to second hand tobacco smoke during pregnancy and well below the mean differences between women who do and do not smoke during pregnancy.

The rate of low birthweight (<2500 grams) was 29% in our most recent trial in this area and the cluster related design effect for the proportion born low birthweight was 2.36. Assuming a Type I error of 5% (2-sided), a 20% treatment effect, and sample size of 1175 enrolled live births before stove installation and an equivalent number after installation, the power was >80% for phase one but only 47% for phase two.

### Ethical review

These trials were reviewed and approved by the Institutional Review Boards at the Johns Hopkins Bloomberg School of Public Health (USA) and the Institute of Medicine at Tribhuvan University (Nepal) and registered at Clinicaltrials.gov (NCT 00786877, Nov. 5, 2008). An independent Data and Safety Monitoring Board (DSMB) was formed and met once prior to the start of the study, once approximately mid-way through the first trial, and is scheduled to meet again in early 2015 to review the results of both trials. The DSMB did not adopt any formal stopping rules or guidelines for efficacy, safety, or futility.

## Conclusion

These trials were designed to address an important public health risk that is especially prevalent in low- and middle-income countries where large proportions of the population use open burning of biomass as their primary household fuel. Phase two was redesigned during phase one when it was clear that reductions in PM concentrations were not as large as anticipated and to provide another point on the dose response curve that was not estimable using commercially available low-cost, biomass stoves. The results of these trials will provide important new information on whether such interventions can impact the most important health outcomes associated with high PM exposure in households and whether scaling programs using currently available technologies is likely to impact health status in these high risk populations. We anticipate results from both phases will be reported in late 2014 or early 2015.
